# X-Linked Lymphoproliferative Syndrome and Common Variable Immunodeficiency May Not Be Differentiated by *SH2D1A* and *XIAP/BIRC4* Genes Sequence Analysis

**DOI:** 10.1155/2011/121258

**Published:** 2011-04-12

**Authors:** Nesrin Gulez, Guzide Aksu, Afig Berdeli, Neslihan Karaca, Sema Tanrıverdi, Necil Kutukculer, Elif Azarsiz

**Affiliations:** Department of Pediatric Immunology, Ege University Medical School, 35100 Izmir, Turkey

## Abstract

The X-linked lymphoproliferative syndrome (XLP) is a rare, inherited immunodeficiency characterized by recurrent episodes of hemophagocytic lymphohistiocytosis, hypogammaglobulinemia, and/or lymphomas. Recently, X-linked inhibitor of apoptosis (*XIAP/BIRC4*) gene defects, in families with XLP but without *SH2D1A* gene defects, has been defined. The distinction from primary immunodeficiencies with a defined genetic cause is mandatory. A six-year-old male patient was admitted with the complaints of persistent general lymphadenopathy, for two years had fever, bilateral cervical multiple microlymphadenopathy, hepatic/splenic enlargement with laboratory findings as decreased serum immunoglobulins, negative EBV VCA IgM (viral capsid antigen) and anti-EBV EA (antibody to early D antigen), positive EBV VCA IgG (viral capsid antigen) and EBV EBNA (antibody to nuclear antigen). *SH2D1A* gene analysis was negative. *XIAP/BIRC4* sequencing revealed two novel single nucleotide variants (exon 7, 1978G > A, and 1996T > A) in the 3′UTR of the gene in both patient and mother which were not disease causing. XIAP protein expression was found to be normal. The clinical and laboratory resemblance, no gene mutations, and normal XIAP protein expression led us to think that there may be another responsible gene for XLP. The patient will to be followed up as CVID until he presents new diagnostic signs or until the identification of a new gene.

## 1. Introduction

The X-linked lymphoproliferative syndrome (XLP) is a rare, inherited immunodeficiency characterized by recurrent episodes of hemophagocytic lymphohistiocytosis (HLH), hypogammaglobulinemia, and/or lymphomas [1]. It is exceptional among human X-linked immunodeficiencies as critical events occur after EBV infection. However, detailed analyses of the affected individuals revealed that the immune defect was broader than the impaired control of EBV infection [2]. Normally, primary EBV infection can occur without characteristic symptoms, or it can elicit mononucleosis of variable severity, but it regularly subsides. On the contrary, in the XLP patients, mononucleosis can be fatal with explosive activation and proliferation of cellular components of the immune system. The life threatening immunological defect is thus characterized by the defect of protection against the proliferation of EBV-transformed B cells [2–4].

Mutations in the signalling lymphocyte activation molecule- (SLAM-) associated protein SAP are responsible for 60–80% of cases of familial XLP [1, 4–6]. The gene defective in XLP has been identified at Xq25 and has been defined as SH2D1A. Mutation analyses of the gene are currently required for a definitive diagnosis of XLP [6]. Recently, mutations in the X-linked inhibitor of apoptosis (*XIAP/BIRC4*) gene, in families with XLP but without SAP mutation, have been defined [1, 7]. Zhu et al. evaluated 42 patients having XLP and identified XIAP deficiency at 2.4% of them [8].

X-linked inhibitor of apoptosis protein (XIAP) has originally been described as an antiapoptotic protein that acts through direct inhibition of caspases. It is ubiquitously expressed in all normal tissues [9–11]. However, XIAP is also involved in a number of signaling pathways, for example, in nuclear factor kB, transforming growth factor, and bone morphogenetic protein signaling pathways [4, 7, 8]. Polymorphisms in the *XIAP/BIRC4 *gene may influence XIAP production and activity [12]. In addition, loss of function due to nonsense mutations in the *XIAP* gene have been observed in patients with XLP [1, 7, 8, 13].

Common variable immunodeficiency (CVID) is the most prevalent symptomatic primary immunodeficiency in humans [13]. Despite the discovery of genetic defects in *ICOS*, *TNFRSF13b/TACI*, and *CD19*, which prove that defects in single genes can be associated with a CVID phenotype, the diagnosis of CVID remains as clinical diagnosis of exclusion in most patients [13]. CVID patients may also have susceptibility to malignancy, especially lymphomas [14, 15]. Several genetically defined primary immunodeficiencies including XLP may mimic CVID.

Based on the previous reports that XLP and CVID may mimic each other, we report a 6-year-old boy, who has the clinical and laboratory signs of both disease. We revealed the difficulty of having an exact diagnosis although molecular analysis for XLP was performed.

## 2. Case Presentation

A six-year-old male patient, second child of nonconsanguineous healthy parents, was admitted to hospital with complaints of persistent general lymphadenopathy, especially at the cervical region, for the last two years. He had one healthy brother. On admission, he was four years old, and his weight was 15 kg (25th percentile) while his height was 112 cm (50–75th percentile). Clinical findings were fever, pallor, ragades on lips, bilateral cervical multiple microlymphadenopathy, grade 2-3 hypertrophic tonsils and pharyngeal hyperemia, and hepatic and splenic enlargement.

The laboratory results were as follows: white blood cell count 14.600/mm^3^ with 26% polymorphonuclear cells, 70% lymphocytes, 4% monocytes on peripheral smear. Acute phase reactants such as CRP concentrations (C reactive protein) and erythrocyte sedimentation rate (ESR) were normal. On admission, he had mildly decreased serum IgG while IgA and IgM concentrations showed normal levels (IgG 694 mg/dL, IgM 84.7 mg/dL, IgA 39 mg/dL) [16]. However, during follow-up of one year, immunoglobulins decreased continuously (pre-IVIG-IgG: 578–495–342 mg/dL, IgM:72–64–32 mg/dL, IgA: 37–32–29 mg/dL). Specific antibody responses (IgG) against tetanus (80 mIU/mL) and haemophilus influenza type B (<1 *μ*g/mL) were inadequate.

Lymphocyte subset analysis revealed the percentages of lymphocytes bearing CD3+; 51%, CD19+; 37%, CD3+CD4+; 17%, CD3+CD8+; 27%, CD19+CD40+; 37%, CD40L; 75%, CD16+CD56+CD3+ NKT (natural killer T cells); 1.8% (183/mm^3^) and CD3−CD56+; 8%. Serological investigations for CMV (Cytomegalovirus), HIV (Human Immunodeficiency Virus), HBV (Hepatitis B virus), HSV (Herpes Simplex virus), Parvovirus, and culture for acid fast bacilli and toxoplasma serology were negative. EBV VCA IgM was negative, and IgG was positive. Paraaortic multiple lymphadenomegalies were shown by abdominal ultrasonography. Direct Coombs test and antinuclear antibody were negative (Table 1).

In addition to low IgG and normal IgM, class switch recombination test (Dr. Anne Durandy's lab, Necker, Paris, France) was found to be absent, and this finding raised the suspicion of possible hyper-IgM syndrome (class switch recombination defects) in the first six months of follow-up. After an episode of fever accompanied by generalized lymphadenopathy, it was found out that he was antiEBV VCA and antiEBV EBNA positive and no EBV EA antibodies were measured. He was positive for EBV DNA on two biopsies from cervical and intraabdominal lymph nodes. Bone marrow examination ruled out myelodysplastic syndrome, lymphohistiocytosis, and lymphoma. When it was confirmed that he had persistent EBV infection accompanied by mild hypogammaglobulinemia, possibility of XLP was searched out.

Initial screening for a mutation in the *SH2D1A* gene was carried out, and no gene defect was identified. Consequently, *XIAP/BIRC4* gene was investigated. Sequence analysis was carried out on genomic DNA extracted from EDTA anticoagulated venous blood using QiAamp DNA Blood Mini Kit (QIAGEN GmbH, Hilden Germany) according to the manufacturer's instructions. All 7 exons of *XIAP/BIRC4 *gene were amplified by polymerase chain reaction (PCR) using flanking intronic primers. PCR amplicons were sequenced in both directions with an ABI 3100 automatic DNA sequencer. Before sequencing, the PCR products were purified using Exo-SAP PCR purification Kit (Amersham Life Sciences).

Seven exons of *XIAP/BIRC4 *gene were searched out, and a heterozygous 1978G > A and 1996T > A nucleotide substitution in region of 3′UTR in exon 7 was detected. Defect in this region of *XIAP/BIRC4 *gene has not been reported elsewhere including INFEVERS database. The same finding was found in the mother while father and elder brother of the patient were normal for *XIAP/BIRC4 *sequencing (Table 2). Then, the samples of the patient were sent to a European center experienced in research of XLP patients, and XIAP protein expression was examined by Western Blot method [1]. Expression of XIAP was found to be normal in the lysate of peripheric blood lymphocytes of the patient.

On the third year of his follow-up in out-patient clinic, he developed multiple enlarged intraabdominal lymphadenomegaly and admitted to Pediatric Emergency Department with the diagnosis of invagination of intestines. After operation, in biopsy specimens, EBV-positive non-Hodgkin lymphoma (Burkitt) was observed (stage III and risk group II). Methotrexate, vincristine, cytarabine, etoposide, and intrathecally methotrexate, arabinoside-C combined therapies were applied and recovered in four months. Now, he is on regular intravenous immunoglobulin treatment and still in follow-up in pediatric immunology out-patient clinic.

## 3. Discussion

Diagnosis of XLP is complicated because of its clinical heterogeneity and rare incidence, particularly in males manifesting with a phenotype consistent with XLP but without a family history. Lymphadenopathy, splenic and/or liver enlargement, fever, dysglammagobulinemia, anemia, and thrombocytopenia are also common features of various immunodeficiencies, including CVID.

Clinical and laboratory findings of our patient were similar to previous XLP reports [1, 7, 13]. All patients, in these series had splenic enlargement. In Salzer et al. series [13], lymph node enlargement was reported in 55% of patients and hypogammaglobulinemia was reported in all of patients as in our case. Rigaud et al. [1] reported hypogammaglobulinemia in only three patients out of 12. They reported HLH in 11 of 12 patients, but in our case and also in other two series, HLH was not present [1, 7, 13].

There was a proven EBV infection in our patient. The presence of EBV infection was 75% in Rigaud et al. [1] and 22% in Salzer et al. [13] reports, respectively. Although colitis was the prominent symptom in Rigaud et al. [1] and Salzer et al. [13] series, it was absent in our and Doshi et al. cases. Rigaud et al. [1] reported the mean age of the patients as six years. Salzer et al. examined sequence analysis of BIRC4/XIAP genes in male patients who were previously diagnosed as CVID; therefore, the mean age of these patients reported in this study was 32 years [13].

NKT cells are a unique population of T cells that express an invariant T cell receptor which recognizes glycosphingolipid antigens presented by the CD1d molecule. These cells are known to be absent in patients with XLP due to *SAP* gene defect [4]. XIAP-deficient patients also show no T, B, or NK cell lymphopenia, but very low numbers of NKT cell [1, 4]. Rigaud et al. [1] thought that NKT cells might be particularly sensitive to apoptosis, and XIAP might be required for their survival and/or development [1]. In contrast, Marsh et al. [17] concluded that invariant NKT cells (iNKT) (defined as CD3 lymphocytes bearing an invariant TcR V*α*14 V*β*11) were not decreased in the majority of XIAP-deficient patients. In our patient, the percentages of total T and NK cells are normal. B cells and CD8+ cells were slightly elevated, probably because of recurrent and chronic infections. NKT cell percentage (defined by the coexpression of CD3 and CD16/CD56) was found to be low by flow cytometric analyses compared to age-matched reference values of healthy children [18–21].

The clinical and some laboratory signs were good evidences to diagnose XLP. However, *SH2D1A* gene was normal, and the finding in 3′UTR region of seventh exon of *XIAP/BIRC4 *gene was not thought to be disease causing, because of reported public databases. In the previous reports, Salzer et al. [13] pointed out that these 3′UTR nucleotide changes are polymorphisms. In addition, the mother who had the same amino acid changes was very healthy. Normal expression of XIAP protein confirmed our suggestions. Furthermore, it is very unlikely that XIAP is involved in the pathology of this patient as no association with lymphoma has been reported yet [22]. The clinical and laboratory resemblance and the findings of no gene mutation and normal XIAP protein expression led us to think that there may be another responsible gene for XLP.

Le Guern et al. [23] described two CVID cases who developed B cell lymphomas, one related to EBV infection, 5 and 12 years after CVID had been diagnosed. Polizzotto et al. [24] reported a case of Burkitt lymphoma in the setting of CVID. Because of the occurrence of lymphomas during the course of CVID, the other diagnosis for our patient is still CVID. This patient also fulfills the criteria for CVID [25]. He will be followed up and managed as CVID until he presents new signs or until the identification of a new gene.

In conclusion, the differential diagnosis is not always easy between XLP and CVID patients. Molecular analysis for well-known mutated genes of XLP may not solve the problem and the patients have to be carefully long-term monitored and treated for life-threatening complications.

## Figures and Tables

**Table 1:** Laboratory findings of patient.

	Patient	Normal values for his age
IgG (mg/dL)	694	986.2 ± 209.6
IgM (mg/dL)	84.7	105.8 ± 40.8
IgA (mg/dL)	39	91.9 ± 37.4
WBC/mm^3^	14600 (26% pnl, 70% lym, 4% mono)	4000–10400
Absolute lymphocyte counts/mm^3^	10220	1500–5200
CD3+ (%-absolute count)	51–5212	55–79/1900–3600
CD19+ (%-absolute count)	37–3781	11–31/300–1200
CD3+CD4+ (%-absolute count)	17–1737	26–49 / 600–2000
CD3+CD8+ (%-absolute count)	27–2759	9–35/300–1300
CD19+CD40+ (%-absolute count)	37–3781	Expressed on all B lymphocytes
CD40L+ on activated T cells (%)	75	50 (24 hours after stimulation)
CD3−CD56+CD16+ (NK) (%-absolute count)	8–817	4–26/90–900
CD3−CD56+CD16+ (NKT) (%-absolute count)	1.8	>4
Anti-nuclear antikor	Negative	Negative
Direct Coombs	Negative	Negative
AntiEBV VCA (IgM)	Negative	Negative
AntiEBV VCA (IgG)	Positive	Negative
AntiEBV EBNA (IgG)	Positive	Negative
EBV anti-EA	Negative	Negative
EBV DNA	Positive (lymph node biopsy)	Negative

**Table 2:** Molecular analysis of *SH2D1A* and *XIAP/BIRC4* genes in patient and family members.

	Patient	Father	Mother	Brother
*SH2D1A*	N	N	N	N
*XIAP/BIRC4*	Heterozygous 1978 G > A and 1996 T > A nucleotide substitution in 3′UTR region of exon7	N	Heterozygous 1978 G > A and 1996 T > A nucleotide substitution in 3′UTR region of exon 7	N
